# Medical Literature

**Published:** 1856-03

**Authors:** 


*Medical Literature.*—We have noticed, of late, a disposition on the part of the more "earnest" and "gushing" minds of the profession, to break over the strict didacticism of medical writing, and to pour into it something of that romantic depth and fervor, that Byronic warmth, which has heretofore been supposed to be somewhat inapplicable to scientific discussion.

We say "of late"—By this we mean that the era of eloquence in medicine is comparatively recent. To be sure, in this, as in all other great improvements, there have been *avant-couriers*, feeble heraldings of the good time coming, warm hues in the orient, prognostic of a glorious day. From the earlier days of medical journalism, it has been customary, in reporting a fatal case, to do the pathetic at the close by stating the metaphorical fact that "death closed the scene," or that "in despite of the most active treatment, the brittle cord of Life was broken"—sometimes the expression "silver cord" was preferred.

When Carlyle made his advent, and virtually declared "all men (who chose to follow his example) free and independent" of the English Grammar, another impulse was given to medical eloquence. Prof. Meigs, of Philadelphia, benefitted largely by this. To those ambitious of a similar success, we would suggest that its secret lies in the position of the adjective. Thus instead of saying "bilious diarrhœa," the Meigsian verbiage would be "profluvium, icterode and melancholic, imperilling the Ego of the organism; the endangium."

But these were only faint indications of the rhetorical and poetic capacity of medicine. Novels began to make their appearance whereof the hero was a Doctor, who suffered multitudinous wrong at the hands of an overbearing neighboring practitioner; and finally, just as the sheriff was about to imprison him for debt, saved by his skill the life of a rich widow, who pays his debts, marries him off to her daughter, and the tale closes by leaving him in the enjoyment of a lucrative practice, honored by the rich and the benefactor of the poor.

At about the same period indignant invective became a special feature of professional writing. The bosoms of certain individuals became so very full of virtuous indignation at the intense ignorance, selfishness and old fogyism of all the older, more wealthy and successful practitioners, that it became imperatively necessary to establish "Scalpels," "Blisters," and other trenchant periodicals, devoted to the especial mission of convincing the people that medicine, as usually practiced by these aforesaid men, was arrant murder, and that only to Young Physic could the people look for honesty or capacity.

Gradually this leaven of improvement has leavened the whole mass of our literature. No one thinks of reporting a case, now-a-days, without informing us that the disease in question was either "truly Protean," or "hydra-headed." "Pandora's box" is a convertible term with general pathology, and is also extremely useful in composition.

Believing, as we do, that this is destined to accomplish a great good in relieving medical writers of the absurd necessity of precision and accuracy of language, and more especially in enabling the young and ambitious writer to do justice to his genius, without imposing upon him the tedious process of studying his subject, and consequently promoting a fair equality among medical writers, so that the mere want of attainment may not be permitted to depress the aspirations of men of talent, who do not happen to be learned, we have been moved to offer to the consideration of young medical writers some models of style, which are eminently worthy of their imitation.

We wish also to suggest that a purely didactic style is not calculated to enlist the enthusiasm, or excite the attention of the student. Our friends of the religious press have got over this by sending out to the world a series of religious novels, in which the truths of the Gospel are so neatly sugared over as to be scarcely recognized. In this form very much sound doctrine is swindled into the unconverted, without their knowledge or consent.

Acting upon this hint we shall suggest, as a very promising labor, the task of inditing a novel, which, under the guise of an allegory, shall unwittingly instruct its readers in the properties and qualities of the indigenous flora of South America. Such a work has been already commenced, and we are enabled, by the kindness of its author, to present to our readers a specimen from its opening chapter. Its fascinating style will beguile the reader, unawares, into an intimate knowledge of the purgatives, deobstruents and emetics of that rich region. But read:

"On the green banks of the Ipecacuanha, near the base of the majestic Pampas, lived in early times a saponaceous Barbican, descended from the royal Serf of ancient Opodeldoc. In his small but comfortable sarabond, composed of green viaticum and aromatic certiorari, this neglected surrogate enjoyed a varicose retirement with his only child, the fair Sarsaparilla. Oft in the stilly night, the traveler, as he crossed the Gutta Percha, or gazed from the summit of Papyrus on the valley of Neuralgia, has heard the voice of this insensate anodyne, as she swept the chords of her bandanna, and poured forth one of the sciatic capsules of her native Gypsum."

From this specimen brick our readers will not fail to recognize the varied and profound knowledge of the materia medica, which the student would derive as he devoured its "thrilling" pages.

Again, poetry must be — has been — called to the aid of our profession. Oliver Wendell Holmes once attempted a medical epic, only a fragment of which has been published. For the subjoined gem we are indebted to Prof. M. It was written at the time he was under conviction of the merits of the speculum, and was engaged in the inquiry as to the relative merits of the "indifferent, antiphlogistic or potential" application of the "caustic pencil." We regret that our space forbids the introduction of more than two stanzas, and those, even, somewhat disconnected:

"Methought I stood upon a cone
Of solid allopathic stone,
And gazed athwart the breezy skies;
When lo, from yonder planisphere,
A vapid atrabilious tear
Was shed by pantomimic eyes.

"Again, again, my bark is tossed
Upon the raging holocaust
Of that acidulated sea;
And diapasons pouring down
With lunar-caustic join to drown
My transcendental epopee!"

We close our illustrative selections with a single verse from a work on general practice, soon to be published:

"HÆMOPTYSIS."

A sensation of weight and oppression at the chest, sirs,
With tickling at the larynx, which scarcely gives you rest, sirs,
Full hard pulse, salt taste, and tongue vei y white, sirs,
And blood brought up in coughing of color very bright, sirs;
It depends on causes three — the first 's exhalation,
The next a ruptured artery — the third ulceration.
In treatment we may bleed, keep the patient cool and quiet,
Acid drinks, digitalis, and attend to a low diet.
Sing hey, sing ho, we do not grieve
When this formidable illness takes its leave.

We trust that the few hints and suggestions thus thrown out will be appreciated. In the possible event that we may, at some future period, be as thoroughly destitute of editorial matter as we now are, we may (but not on any other terms) continue our selections.

